# Seroprevalence and Associated Risk Factors of Rift Valley Fever in Domestic Small Ruminants in the North Region of Cameroon

**DOI:** 10.1155/2019/8149897

**Published:** 2019-11-25

**Authors:** R. Poueme, F. Stoek, N. Nloga, J. Awah-Ndukum, M. Rissmann, A. Schulz, A. Wade, J. Kouamo, M. Moctar, A. Eisenbarth, L. God-yang, S. Dickmu, M. Eiden, M. H. Groschup

**Affiliations:** ^1^Laboratoire National Vétérinaire, Garoua, Cameroon; ^2^Faculty of Sciences, University of Ngaoundere, Cameroon; ^3^Institute of Novel and Emerging Infectious Diseases, Friedrich-Loeffler-Institut, Greifswald–Insel Riems, Germany; ^4^School of Veterinary Medicine and Sciences, University of Ngaoundere, Cameroon

## Abstract

Rift Valley fever (RVF) is a zoonotic vector borne infectious disease of major medical and veterinary importance particularly in sub-Saharan Africa. However, there is dearth of epidemiological knowledge of the disease in Cameroon. We conducted a cross-sectional study (January 2016–January 2017) to investigate the seroprevalence and potential risk factors of Rift Valley fever virus (RVFV) in sheep and goats in the North region of Cameroon. Stratified sampling approach was used to select herds where sera were collected from 680 randomly selected small ruminants (355 goats and 325 sheep) in eight localities (Kismatari, Lagdo, Pitoa, Garoua, Bocklé, Dembo, Poli and Touboro) within three administrative divisions (Bénoué, Mayo-Rey and Faro) in the North region. Anti-RVFV antibodies were detected using a competitive Enzyme-Linked Immunosorbent Assay (ELISA), while a capture ELISA was used for the detection of specific RVFV-Immunoglobulin M (Ig-M) antibodies. We evaluated the associated potential risk factors of RVF in small ruminants based on observations of animal-related intrinsic and extrinsic factors in combination with serological results. The results revealed that 3.4% (95% confidence interval (CI): 2.2–5.1%) of sampled animals and 24.6% (95% CI: 15.1–37.1%) of 65 sampled herds were seropositive for anti-RVFV antibodies and no difference in seropositivity between sheep and goats at individual animal as well as at herd levels was observed. Localities along hydrographic or large water banks such as Kismatari (OR: 14.333, (95% CI: 1.436–145.088)) and Pitoa (OR = 11.467 (95% CI: 1.249–50.306)) were significantly associated to RVFV antibody seroprevalence in a simple logistic regression. In addition, the multiple regression analysis showed that age and access to water points significantly influenced RVFV antibody seroprevalence in small ruminants. This study revealed that anti-RVFV antibodies are present in sheep and goats in the North region of Cameroon. It highlights the likely endemic circulation of RVFV in the considered localities despite the absence of clinical cases reported in animals or humans. Under these conditions, it is necessary to set up an early warning, surveillance and control strategy based on epizootic risk.

## 1. Introduction

Rift Valley fever (RVF) is an infectious disease of many wild and domestic animal species [1, 2] caused by a RNA virus belonging to the order Bunyavirales, family *Phenuiviridae*, genus Phlebovirus [3, 4]. In ruminants, Rift Valley fever virus (RVFV) causes abortions and a high mortality range of up to 80–100% in newborn animals [5]. The disease is transmitted by mosquitoes of several genera, including *Aedes spp*., and *Culex spp*., [6, 7]. RVFV is of major medical and veterinary importance due to its large geographical spread. In the past, several epizootics and epidemics were recorded in sub-Saharan Africa [8, 9]. First described in 1931 as massive abortions and necrotic hepatitis in sheep in the Rift Valley of Kenya [10, 11], this zoonotic infection has also been observed in humans [12]. Breeders, veterinarians, livestock assistants, slaughterhouse staff and butchers are particularly at risk and often infected by direct or indirect contact with blood, body fluids and organs of infected animals [4, 12]. The common signs observed in humans are mild flu-like manifestations with fever, myalgia, headache and arthralgia, whereas severe cases can also develop retinitis, encephalitis and hemorrhagic fever [13]. Human infections due to mosquito bites have also been reported [14].

After the initial report of the disease [10, 11], RVF was observed in most countries in South and East Africa (e.g., Kenya and South Africa) [11] with major epidemics occurring almost every 15 years generally after heavy rainfall in the respective area [15–17]. Climatic and environmental factors in East Africa are used to predict epidemics, nevertheless the pattern observed in 1987 in the Senegal River basin could not be explained by these factors [18]. For example, RVF outbreaks were observed in southern Mauritania (1982–1985) during periods of severe drought with no rain [19]. The spread of the virus in Barkedji (Senegal) and Mauritania was rather linked to movement of animals and their concentration around the scanty water points with high vector prevalence [15–17, 19, 20].

RVF was described for the very first time by Maurice in sheep and wild animals (gazelle, buffalo) in North Cameroon in 1967 [21]. A seroprevalence of 22–45% was recorded at those days utilizing a haemagglutination inhibition assay (HI). Subsequent studies on domestic ruminants revealed a RVFV antibody seroprevalence of 9.33% [22] and 13.5% [23] in cattle and 12.28% [22] and 3.4% [23] in small ruminants for northern regions (Far North, North and Adamawa) and 23.07% in goats in the Centre region of Cameroon [24]. RVF is a zoonotic disease and can cause enormous economic loss [25]. Anti-RVFV antibodies have also been detected in humans (1.06%) in southern parts of the country [26], suggesting a virus circulation throughout the entire country.

The northern regions of Cameroon are inhabited by many wild ruminant species which are potential RVFV reservoir hosts and which interact highly with domestic animals [21]. These regions are characterized by very irregular rainfalls with frequent flooding during rainy seasons [27]. During the dry season, temporary pools and irrigation-based farming systems provide favourable conditions for the proliferation of RVFV vectors. These factors highlight the risk of occurrence and epizootic outbreaks of RVF in Cameroon.

Several studies found Cameroon to be at risk of RVFV and prove its low-level local circulation. These results emphasize the need of continuous and extended surveys in Cameroon. In addition, the small ruminant husbandry system in the North region favours the cohabitation of animals with their owners and could enhance the zoonotic risk of transmission. Therefore, this study was conducted to estimate the seroprevalence and to evaluate the potential risk factors for the spread of RVFV in small ruminants in the North region of Cameroon.

## 2. Material and Methods

### 2.1. Description of Study Areas

**Figure 1:**
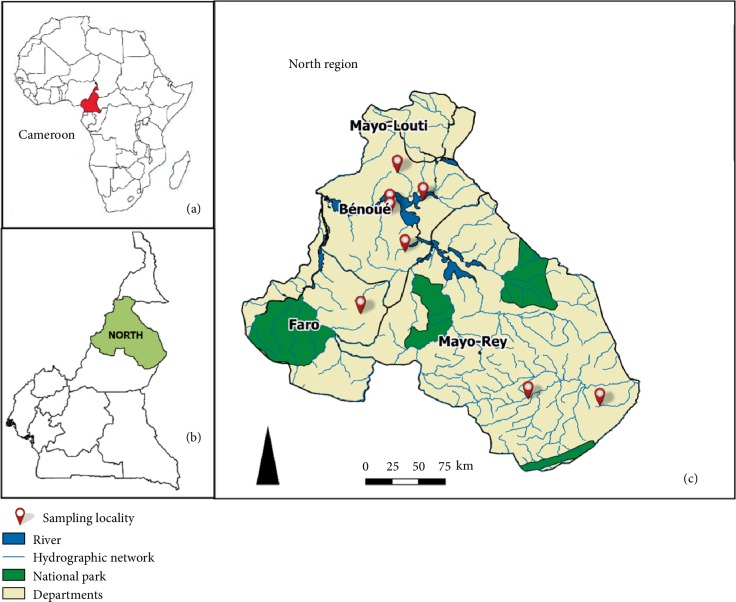
Map showing Cameroon in Africa, the study localities and sampling points in the North region of Cameroon. (a) An insert of Africa map showing Cameroon; (b) an insert of Cameroon map highlighting the North region; (c) extract map showing the study localities and sampling points in the North region of Cameroon.

This study was carried out in eight localities (Lagdo, Pitoa, Bokle, Garoua, Kismatari, Poli, Touboro and Dembo) of three administrative divisions (Bénoué, Mayo-Rey and Faro) of the North region of Cameroon (6°–10°LN and 12°–16°LE) (Figure 1). The North region is situated in the Sudano-Sahelian region, in low to medium altitude areas of the country (average altitude: 249 m) with short rainy seasons from mid-March to October, an annual rainfall range of 1200–1600 mm and an ambient temperature range of 21–36°C. The region is also characterized by the presence of numerous hydrographic networks including the large and long river Bénoué and a large hydroelectric dam in Lagdo. The agricultural systems around these water points are based on irrigation providing good conditions for mosquitoes' development. The communities of the North region in Cameroon are pure pastoralists (30%) and agro-pastoralists (65%), practicing predominantly the traditional systems of husbandry. The region is a major producing zone of small ruminants in Cameroon and the socio-economic, political, cultural and religious activities of the farmers depend almost entirely on livestock.

### 2.2. Selection of Animals for the Study

A cross-sectional study was carried out during the period of January 2016 to 2017 using a stratified sampling procedure to select herds and a random sampling approach for individual small ruminants within the herds. Sampling sites were selected based on relative proportions of small ruminant herds as recorded by veterinary officers of the Divisional Delegations of MINEPIA (Ministry of Livestock, Fisheries and Animal Industries) in the North region and the willingness of the community to participate in the study. A minimum number of 380 domestic small ruminants to be sampled for the whole study area regardless of the species was estimated using the formula [28]:

(1) $$\begin{array}{c} N\;=\;\frac{1.96^{2}\;\times \;P\left(1-P\right)}{d^{2}} \end{array}$$

*N* = estimated minimum sample size; *P* = estimated prevalence (45%, the prevalence reported in small ruminants by Idrissou [22] in northern regions of Cameroon); *d* = precision of 5% (with 95% confidence interval).

In the selected communities, 45% of small ruminants per herd were humanely captured, restrained to avoid suffering and subsequently blood was sampled. Information regarding location, sex, age and herd sizes of the animal, as well as the access to water points were noted. The age of the animals was provided by the farmers or otherwise determined by dental inspection [29]. Nursing and recently weaned kids and lambs (usually less than 5–6 months old) were excluded from the study due to the possible presence of maternal antibody [30]. Animals aged 1–3 years old were considered as producing adults while more than 3 years old animals were considered to be at the end of their production life span. A total of 65 herds including 28 herds of sheep (325 heads) and 37 herds of goats (355 heads) from the eight localities in the study were sampled.

### 2.3. Blood Sampling and Laboratory Analysis

Apart from procedural restraining manipulations for safety purposes and jugular vein puncture for blood sampling (≤5 ml) using sterile vacutainer, the animals were not subjected to suffering. The tubes were labelled with species code and ordered number, then placed in boxes in upright positions until the blood clotted and sera were harvested in 1.5 ml collection tubes. Sera were shipped in an ice box with frozen ice packs to the National Veterinary Laboratory (LANAVET) of Boklé-Garoua, Cameroon where they were kept at −20°C until analysis.

### 2.4. Screening of Antibodies against RVFV

A Competitive Enzyme-Linked Immunosorbent Assay (C-ELISA) (IDvet® ID Screen Rift Valley Fever Competition Multi-species, Grabels, France) for the detection of IgG and IgM antibodies against the nucleoprotein (NP) of RVFV was performed according to the manufacturer's instructions. Briefly, the test was conducted in 96-well polystyrene plates that were precoated with a recombinant RVFV-NP. Test samples and controls were added to the microwells. The anti-NP antibodies in the serum formed an antigen-antibody complex which masked the NP epitopes. An anti-nucleoprotein-peroxidase conjugate (Po) was added to the microwells to bind to free NP epitopes and form an antigen-conjugate-peroxidase complex. After washing in order to eliminate excess conjugate, the substrate solution was added and finally after incubating, the stop solution was added and the absorbance was measured. The inhibition rate was calculated according to the following formula:

(2) $$\begin{array}{c} \frac{S}{N}\;\left(\%\right)\;=\;\frac{{\text{OD}}_{\text{sample}}}{{\text{OD}}_{\text{NC}}}\;\times \;100 \end{array}$$

OD: optical density. NC: negative control. *S*/*N*: competition percentage. *S*/*N* values lower than or equal to 40% were considered positive, values above 50% negative and values in between inconclusive.

### 2.5. Specific IgM Detection

All samples tested positive in the C-ELISA were re-analyzed using the IgM capture ELISA (IDvet® ID Screen Rift Valley Fever IgM Capture, Grabels, France) according to the manufacturer's instructions to specifically detect IgM antibodies. Briefly, the wells were coated with polyclonal anti-ruminant IgM antibody to immobilize IgM in the test sera. After washing, RVFV-NP was added, followed by more washing steps and finally peroxidase-labelled anti-RVFV-NP antibody. The presence of RVFV-specific IgM was revealed eventually by colour reaction. The inhibition rate was calculated according to the following formula:

(3) $$\begin{array}{c} \frac{S}{P}\;\left(\%\right)\;=\;\frac{\text{Net}\;\text{OD}\;\text{of}\;\text{the}\;\text{sample}-\text{Net}\;\text{OD}\;\text{of}\;\text{Negative}\;\text{control}}{\text{Net}\;\text{OD}\;\text{of}\;\text{Positive}\;\text{control}-\text{Net}\;\text{OD}\;\text{of}\;\text{Negative}\;\text{control}}\times 100 \end{array}$$

*S*/*N* values above 50% were considered positive, values lower than or equal to 40% negative and values in between inconclusive.

### 2.6. Statistical Analysis

The data were analysed using Statistical Package for Social Sciences (SPSS) software (IBM SPSS Statistics for Windows, Version 20.0. Armonk, NY: IBM Corp. published in 2011). Descriptive statistics were performed to summarize seroprevalence; 95% confidence intervals were calculated using the Wilson method with continuity correction. The simple logistic regression was used to determine potential risk factors with their respective odds ratios and 95% confidence intervals. After that, multiple logistic regression was performed including potential risk factors with *p* ≤ 0.20. The initial model was reduced stepwise and the final model included the variables “Age” and “Access to water points”. The significance level was set at *p* < 0.05.

## 3. Results

The seroprevalences of anti-RVFV antibodies in small ruminants in the North region of Cameroon stratified by risk factors are summarised in Table 1. The study showed that 23 out of 680 (3.4%, 95% CI: 2.2–5.1%) individual animals were anti-RVFV antibody seropositive while 16 of 65 herds (24.6%; 95% CI: 15.1–37.1%) had at least one seropositive animal and no difference in RVFV antibody seropositivity between sheep and goats at individual animal level and herd level was observed, respectively.

The simple logistic regression indicated that (1) small ruminants in the localities of Kismatari (OR = 14.333; *P* = 0.023) and Pitoa (OR = 11.467; *P* = 0.031) had significantly higher seropositivity to anti-RVFV antibodies than those in other localities, (2) the sex of the animals was not significantly associated to RVFV seropositivity, and (3) the RVFV antibody seroprevalence was not significantly associated with the season (Table 1).

The multiple logistic regression has generated a final model including the variables “Age” and “Access to water points” (Table 2). The *R*^2^ value was estimated at 0.201, which means that the model obtained explains only 20.1% of the observed variability. However, in both, the simple and multiple regression analysis (1) animals along river banks or with access to rivers, ponds and other temporary or permanent water sources had significantly higher seroprevalence of anti-RVFV antibodies compared to those being in very little or no contact with water bodies (OR = 0.158, *p* < 0.0001), and (2) animals within the category of more than 36 months old had higher RVFV seropositivity than their counterpart younger animals (Tables 1 and 2).

The study revealed further that all samples tested positive in the C-ELISA were negative in the IgM capture ELISA.

## 4. Discussion

The study revealed IgG antibodies against RVFV in sheep and goats in the North region of Cameroon indicating that RVFV may be endemic in this region. This implies the most likely but unprovable assumption that positive animals were native and never transported to this area. The overall RVFV antibody seroprevalence found was 3.4% in this study, which is consistent with previous reports [23] for domestic small ruminants (sheep and goats) in the Bénoué division. However, the seroprevalence was lower than the 9.8–20% reported earlier for domestic ruminants in the North region of Cameroon [21, 22, 31] and 10–22% in domestic ruminants in Chad [21, 32] with almost similar climatic conditions. Notwithstanding, this study and previous reports [21–24] highlight the presence of anti-RVFV antibodies in Cameroon, suggesting a possible silent circulation of RVFV with subclinical infections in the North region of Cameroon.

Many factors that reveal the presence of RVFV and the risk for epizootic outbreaks exist in the country. Likewise, anti-RVFV antibodies in domestic and wild animals (gazelle, buffalo) have been reported in neighbouring Chad and other parts of Cameroon [21]. There are several hydrographic conditions and abundant climatic and seasonal events (such as abundant rainfalls, floods, irrigation farming systems) in the studied region (as well as in the entire country) which favour the abundance of mosquitoes.

The study showed age-related effects on RVFV antibody seroprevalence in small ruminants. Sheep and goats have shorter productive life spans (averagely 3-4 years) than cattle (3–5 years for males and >9 years for females) in the current studied region. The present study revealed that particularly old (≥36 months) animals have increased odds of being seropositive than younger animals. This agrees with previous studies of LeBreton et al. [24] in Cameroon, Olaleye et al. [36] in Nigeria, Ringot et al. [32] in Chad, Jeanmaire et al. [37] in Madagascar, Thiongane et al. [38] in Senegal and Sumaye et al. [39] in Tanzania. In addition, the increase in RVFV antibody seroprevalence with age has been observed to be a typical feature of endemic diseases in any geographic region [40].

The present study also reveals that localities and access of animals to water bodies significantly influenced seroprevalence of RVFV in domestic small ruminants. The increased odds of seropositivity found in the simple regression analysis in Kismatari and Pitoa compared to the other localities could be associated with differences in climatic and environmental conditions. Kismatari and Pitoa are situated along the river Bénoué. These riverine communities also practice marshy agriculture (rice growing and onion cultivation) based on irrigation systems that are favourable for the lifecycle of RVFV vectors compared to the dryer environments of the other localities particularly in the Faro and Mayo-Rey divisions. Similar observations have been reported by Ndione et al. [18] who reported the Senegal river basin being a major risk area for RVF viral activity. Humid environments and hydro-agricultural development sites provide favourable conditions for the proliferation of RVFV vectors and thereby an increased risk for maintaining RVFV in the environment [41]. The study showed that animals, which had access to water bodies, have increased odds of being seropositive. Waterholes have long been noted as essential breeding sites for the larval and adult stages of RVFV mosquito vectors [18]. It is likely that RVFV is silently circulating in the localities of the studied region. In agreement with the seroprevalence recorded in this study, Kézié [42] reported a higher RVFV seroprevalence (10.7%) in more humid localities in the Togolese plateau region with large hydrographic networks.

No difference was observed between RVFV antibody seroprevalence in small ruminants during the dry and rainy season in the present study, which is in contrast with Zeller et al. [19] who reported frequent outbreaks and high prevalence during the long dry seasons in Mauritania (1982–1985).

The capture ELISA for specific detection of IgM did not reveal RVFV-IgM antibodies in animals tested positive in the C-ELISA before. This result corroborates those of Rissmann et al. [23], who also did not detect RVFV-IgM antibodies in small domestic ruminants in northern parts of Cameroon. The finding indicates that there were no recent RVFV infections during the sampling period. In general, RVFV-IgM antibodies can be detected in the host for a maximum duration period of two months [30, 43] and are specific markers for a starting or ongoing epidemic. Therefore, a continuous surveillance of small ruminants is highly recommended.

## 5. Conclusion

The study revealed the presence of anti-RVFV antibodies in small ruminants, suggesting that RVFV may be circulating in the North region of Cameroon even though no IgM antibodies were detected. Access of animals to water bodies and age of the animal were associated with RVFV antibody seroprevalence. However, no specific control program exists at national level for RVF in Cameroon. The prevalence and risk analysis of RVFV in animals and humans in Cameroon are understudied and the hazard of RVFV infections is increasingly becoming a major concern particularly for the veterinary and medical services. In order to determine the impact and control measures of RVFV in Cameroon, broad multidisciplinary investigations (One Health Approach) need to be conducted on the potential sources, reservoir hosts and vectors of the virus as well as the routes of transmission, associated risk factors and epidemiology of RVF.

## Figures and Tables

**Table 1:** Seroprevalence of RVFV-specific IgG antibodies in small ruminants in the North region of Cameroon stratified by potential risk factors.

Risk Factor	Variables	Sheep		Goats		Total animals			Odds ratio		
		Examined (Positive)	Prevalence (95% CI)	Examined (Positive)	Prevalence (95% CI)	Examined (Positive)	Prevalence IgM & IgG (%) (95% CI)		OR	95% CI	*P*-value
Division	Bénoué	251 (14)	5.6 (3.2–9.4)	179 (8)	4.5 (2.1–9)	430 (22)	5.1	3.3– 7.7	6.956	0.929–52.110	0.059
	Faro	30 (0)	0	90 (0)	0	120 (0)	0	0	0	0	0
	Mayo Rey	44 (1)	2.3 (0.1–13.5)	86 (0)	0	130 (1)	0.8	0–4.9	/	/	

Localities	Bocklé	59 (3)	5.1 (1.3–15.1)	38 (1)	2.6 (0.1–15.4)	97 (4)	4.1	1.3–10.8	5.548	0.610–50.450	0.128
	Dembo	22 (0)	0	28 (1)	3.6 (0.2–20.3)	50 (1)	2.0	0.1–12	2.633	0.161–42.916	0.497
	Garoua	24 (1)	4.2 (0.2–23.2)	4 (0)	0	28 (1)	3.6	0.2–20.3	4.778	0.290–78.779	0.274
	Kismatari	21 (3)	14.3 (3.8–37.4)	9 (0)	0	30 (3)	10.0	2.6–27.7	14.333	1.436–143.088	0.023
	Lagdo	95 (4)	4.2 (1.4–11)	81 (5)	6.2 (2.3–14.5)	176 (9)	5.1	2.5– 9.8	6.952	0.870–55.577	0.068
	Pitoa	30 (3)	10 (2.6–27.7)	19 (1)	5.3 (0.328.2)	49 (4)	8.2	2.7–20.5	11.467	1.249–105.306	0.031
	Poli	30 (0)	0	90 (0)	0	120 (0)	0	0	0	0	0
	Touboro	44 (1)	2.3 (0.1–13.5)	86 (0)	0	130 (1)	0.8	0–4.9	/	/	/

Season	Dry	212 (13)	6.1 (3.4–10.5)	138 (6)	4.3 (1.7–9.6)	350 (19)	5.4	3.4–8.5	/	/	/
	Rainy	113 (2)	1.8 (0.3–6.9)	217 (2)	0.9 (0.2–3.6)	330 (4)	1.2	0.4–3.3	0.938	0.406–2.170	0.881

Access to water bodies	Yes	166 (13)	7.8 (4.4–13.3)	90 (5)	5.6 (2.1–13.1)	256 (18)	7	4.3–11	/	/	/	
	No	159 (2)	1.3 (0.2–5)	265 (3)	1.1 (0.3–3.5)	424 (5)	1.2	0.4–2.9	0.158	0.058–0.430	<0.0001

Species	Sheep	325 (15)	4.6 (2.7–7.6)	/	/	325 (15)	4.6	2.7–7.6	/	/	/	
	Goats	/	/	355 (8)	2.3 (1.1–4.6)	355 (8)	2.3	1.1–4.6	0.476	0.199–1.139	0.096

Age (months)	≤12	127 (5)	3.9 (1.4–9.4)	148(0)	0	275 (5)	1.8	0.7–4.4	0.157	0.053–0.462	0.001
	12–36	131 (3)	2.3 (0.6–7.1)	170 (4)	2.4 (0.8–6.4)	301 (7)	2.3	1–4.9	0.201	0.076–0.534	0.001
	≥36	67 (7)	10.4 (4.6–20.9)	37 (4)	10.8 (3.5–26.3)	104 (11)	10.6	5.7–18.6	/	/	/

Sex	Male	87 (1)	1.1 (0.1–7.1)	103 (1)	1.0 (0.3–3.1)	190 (2)	1.1	0.2–4.2	/	/	/	
	Female	238 (14)	5.9 (3.4–9.9)	252 (7)	2.8 (1.2–5.9)	490 (21)	4.3	2.7–6.6	4.209	0.977–18.128	0.054

*Total*		325 (15)	4.6 (2.7–7.6)	355 (8)	2.3 (1.1–4.6)	680 (23)	3.4	2.2–5.1	/	/	/	

/: Modality considered as reference while performing logistic regression.

**Table 2:** Parameters of final model obtained after the multiple logistic regression.

Variables in the final model	Coefficients in the equation	S.E.	Wald	df	*P*-value	OR	95% CI for OR	
							Lower	Upper
Age (months) (12–36)	−1.843	0.560	10.828	1	0.001	0.158	0.053	0.475
Age (months) (≥36)	−1.301	0.511	6.488	1	0.011	0.272	0.100	0.741
Access to water bodies (Yes)	−1.785	0.521	11.722	1	0.001	0.168	0.060	0.466
Constant	3.293	0.530	38.543	1	<0.0001	26.910		

OR: odds ratio; d: degree of freedom; CI: confidence interval.

## Data Availability

The raw data used to support the findings of this study are available from the corresponding author upon reasonable request.
